# Quantifying the cell morphology and predicting biological behavior of signet ring cell carcinoma using deep learning

**DOI:** 10.1038/s41598-021-03984-4

**Published:** 2022-01-07

**Authors:** Qian Da, Shijie Deng, Jiahui Li, Hongmei Yi, Xiaodi Huang, Xiaoqun Yang, Teng Yu, Xuan Wang, Jiangshu Liu, Qi Duan, Dimitris Metaxas, Chaofu Wang

**Affiliations:** 1 Department of Pathology, Ruijin Hospital, Shanghai Jiaotong University School of Medicine, Shanghai, China; 2 Sensetime Research, No. 1900 Hongmei Road, Xuhui District, Shanghai, China; 3 Shanghai Pulmonary Hospital, Tongji University School of Medicine, Shanghai, China; 4 Department of Computer Science, Rutgers The State University of New Jersey, Newark, USA

**Keywords:** Gastrointestinal cancer, Machine learning

## Abstract

Signet ring cell carcinoma (SRCC) is a malignant tumor of the digestive system. This tumor has long been considered to be poorly differentiated and highly invasive because it has a higher rate of metastasis than well-differentiated adenocarcinoma. But some studies in recent years have shown that the prognosis of some SRCC is more favorable than other poorly differentiated adenocarcinomas, which suggests that SRCC has different degrees of biological behavior. Therefore, we need to find a histological stratification that can predict the biological behavior of SRCC. Some studies indicate that the morphological status of cells can be linked to the invasiveness potential of cells, however, the traditional histopathological examination can not objectively define and evaluate them. Recent improvements in biomedical image analysis using deep learning (DL) based neural networks could be exploited to identify and analyze SRCC. In this study, we used DL to identify each cancer cell of SRCC in whole slide images (WSIs) and quantify their morphological characteristics and atypia. Our results show that the biological behavior of SRCC can be predicted by quantifying the morphology of cancer cells by DL. This technique could be used to predict the biological behavior and may change the stratified treatment of SRCC.

## Introduction

Signet ring cell carcinoma (SRCC) is an adenocarcinoma with a high degree of malignancy, which occurs most frequently in the stomach and colorectum. SRCC is defined as a tumor consisting mainly or entirely of signet ring cells which are characterized by mucus in the cytoplasm that squeezes the nucleus to one side^1–3^. The pathological diagnosis of SRCC is difficult. Unlike nested squamous cell carcinoma or ductal adenocarcinoma, SRCC cancer cells are diffuse and lack structures that can be recognized at low magnification, and the cell morphology is very similar to plasma cells, intestinal metaplasia, or capillary endothelium. As a result, even the most experienced pathologists or the latest algorithms are still prone to missed diagnosis^1–3^.

With the development of computer technology and advances in DL, clinical-grade digital pathology has become more available for cancer diagnosis^4–7^. Some segmentation algorithms have been successfully developed to detect cell and subcellular levels structure^8–10^. Previously, we proposed a novel detection framework of signet ring cell based on semi-supervised learning from whole slide images (WSIs)^11^. By combining self-training and cooperative training, our DL model utilizes labeled and unlabeled data better, and experiments on a large number of real clinical data have proved its effectiveness. As far as we know, this is the only scheme that can use DL to detect signet ring cells automatically and accurately.

In this study, we used the model to analyze a total of 607 WSIs. More than 29 million cells were detected. Primary site of these tumors mainly includes the stomach (42.7%) and colorectum (56.8%). We not only analyzed the tumor cells of the primary lesion but also identified and analyzed metastatic regional lymph nodes, peritoneal implants, and ovarian implant tumor, namely the Krukenburg tumor. For this task, four features have been extracted from images: cell cross-sectional area, nuclear area, ellipticity, and nuclear/cytoplasmic ratio. Our results show that the inherent properties and differences of these cells are related to the depth of tumor invasion and the mode of metastasis, and can predict biological behavior to some extent. We believe that our results reveal important information to render an accurate diagnosis and thus, more accurate treatment strategies for the treatment of SRCC.

## Results

### Overview of the datasets

**Figure 1:**
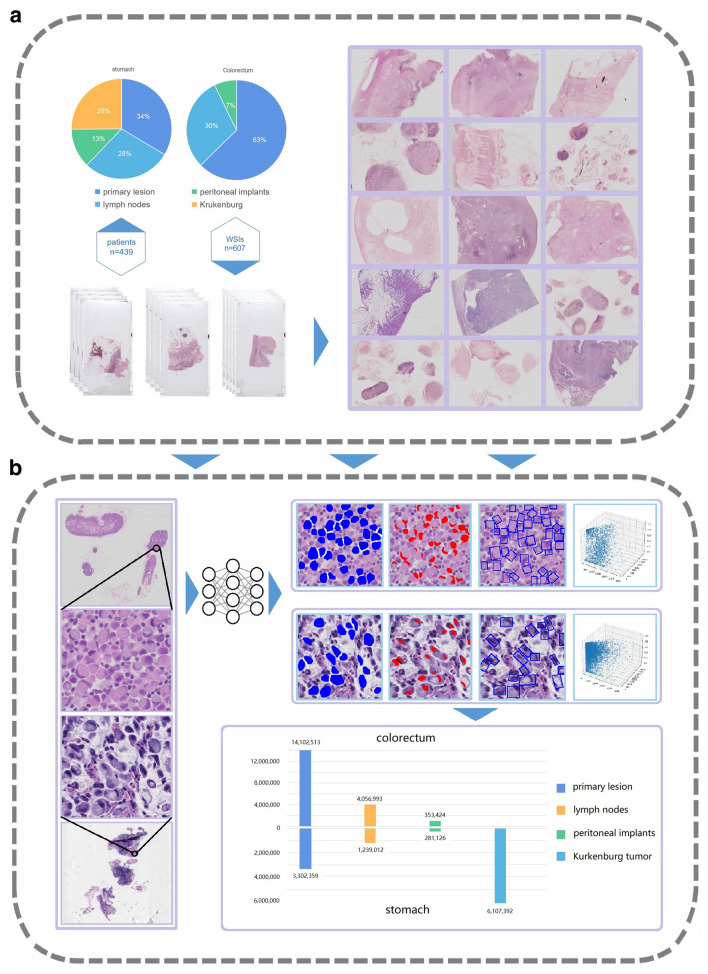
Workflow of data collecting, scanning and analyzing. (**a**) The datasets consists of 607 WSIs that were collected from 439 patients. After summarizing each dataset, the HE slides were scanned to obtain WSIs. (**b**) WSIs were then analyzed by our DL model. Visualization results provided by DL, including the cross-sectional area of cell and nuclear, and the minimum circumscribed rectangle (representing the ellipcity), were illustrated. The 3D-scatter plot represents the inherent property distribution of each cell in a WSI. Over 29 million cells were detected and analyzed.

**Figure 2:**
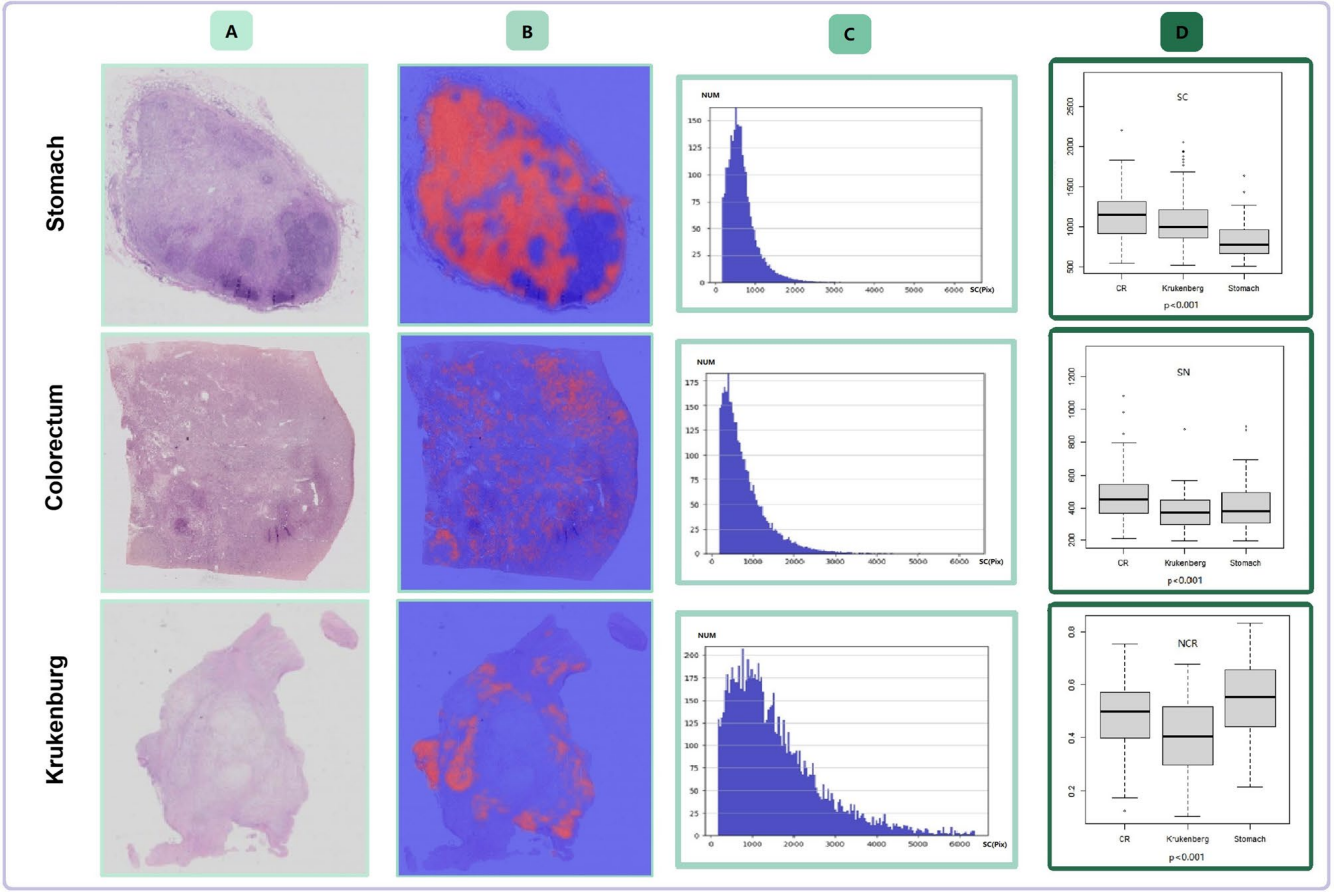
Comparison of cell properties. Each cell in each WSI was analyzed to determine the inherent properties of the signet ring cells in the case. (**A**) digital slices obtained by scanner (**B**) highlights the signet ring cells in heatmap (**C**) the distribution of cell number and cross-sectional area was shown on the histogram (**D**) the boxplot shows the comparison of inherent properties of all cases (Independent sample t test).

A total of 607 whole-slide images (WSIs) extracted from high resolution were analyzed. More than 29 million cells were quantified by the deep-learning framework we developed (Fig. 1). Four inherent cell properties were measured: average cell area (SC), nucleus area (SN), ellipticity (EP), and nuclear-plasma ratio (NCR). The standard deviation of each term is used to represent the atypia. According to the primary site, these sections were divided into stomach (259, 42.7%) and colorectum (348, 56.8%). Except for the primary tumor (87/259, 33.6%; 218/348, 62.6%), we also analyzed the metastatic regional lymph nodes (74/259, 28.6%; 105/348, 30.2%), peritoneal cancer nodules (33/259, 12.7%; 25/348, 7.2%), and ovarian implant metastatic lesions, namely Krukenburg tumor (65/259, 25.1%) (Fig. 2 and Supplementary Table 1).

### Biological behavior and cell morphology of gastric SRCC

**Figure 3:**
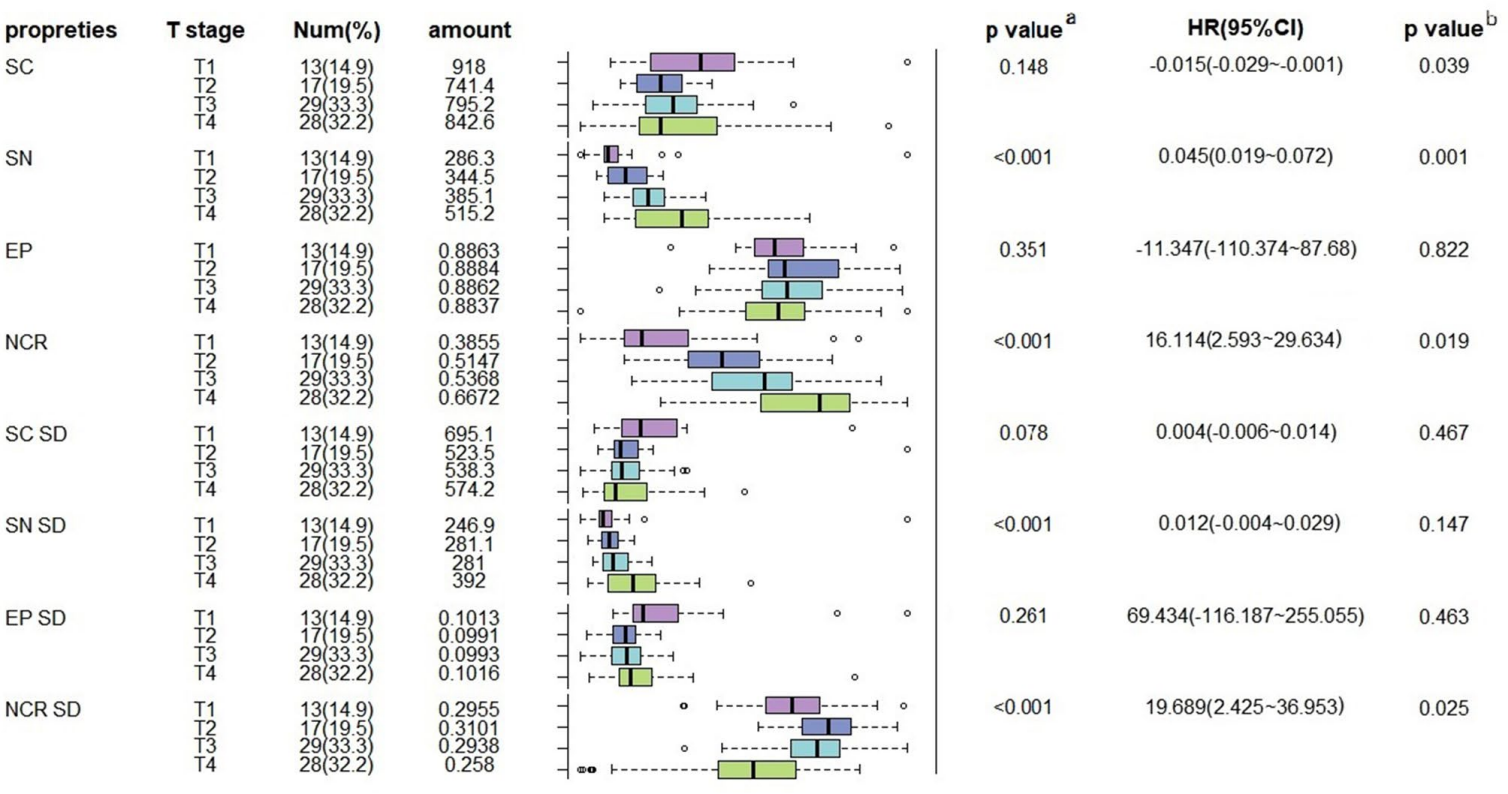
Univariate and multivariate analysis of the relationship between lymph node involvement and the depth of invasion in stomach SRCC. SC Cell area (pixel); SN Nucleus area (pixel); Ep Ellipticity; NCR Nuclear-plasma ratio; SD Standard deviation. #a calculated by Kruskal–Wallis test; b calculated by multiple ordered logistic regression, *p* < 0.05 was considered to be significant.

Gastric SRCC is a special entity of poorly differentiated adenocarcinoma in the pathological classification of gastric cancer. The prognosis of gastric SRCC in different TNM stages is significantly different: early gastric SRCC has a low rate of lymph node metastasis and a better prognosis than other types of early gastric cancer, while advanced gastric SRCC has high invasion and metastatic ability and poor prognosis^1–3^. Early gastric SRCC can be cured by endoscopic submucosal dissection (ESD) or endoscopic mucosal resection (EMR) to reduce postoperative complications^12^. Here we analyze the condition of gastric SRCC. This part of the analysis cohort consists of 87 WSIs, which can be divided into either lymph node involvement or not, or T1 ~ T4 according to the depth of invasion according to the current AJCC TNM staging system (Supplementary Table 1). We found that SN and NCR as well as their atypia was most correlated with the T stage (*p* < 0.001), while the size or regular degree of tumor cells are not. This suggests that the morphology of the nucleus can be a predictor of the depth of tumor invasion. Besides, the results of the multivariate analysis show that SN, NCR, and NCR SD are independent significant predictors of the T stage in gastric SRCC. Tumors with larger nuclei (HR 0.045, 95%CI 0.019–0.072, *p* = 0.001) and bigger nuclear-plasma ratio (HR 16.144, 95%CI 2.593–29.634, *p* = 0.019) tend to have a higher T stage, while as the tumor infiltrates deeper, the atypia of nuclear-plasma ratio gets lower (HR 19.689 95%CI 2.425–36.953, *p* = 0.023) (Fig. 3). This result suggests that there is significant heterogeneity in the nucleus morphology of gastric SRCC. Our results combined with gastroscopic biopsy may be helpful to predict the depth of tumor invasion, evaluate the prognosis of patients, and even guide treatment for patients to choose appropriate treatment. Our results coincide with the Phillip et al.^13^ conclusion that the shape of nucleus can encode prognostic information for different types of cancer.

Lymph node metastasis is required to assess in pathologic staging of gastric SRCC for its prognostic value of postoperative patients. However, most studies believe that there is no reliable approach to predict gastric SRCC lymph node metastasis before the operation, even with the tissues from sentinel node biopsy^14^, *which becomes one of the reasons for overtreatment*. Our study revealed that there was a statistically significant correlation between the ellipticity difference and lymph node metastasis in gastric SRCC. The cells in the lymph node-positive group tend to have a larger EP SD than the lymph node-negative group (0.1013:0.0991, *p* = 0.045) (Supplementary Table 2), which means that their shapes are more irregular. These results suggest that it is possible to predict lymph node metastasis by cell morphology, which depends on the physical properties of the cell membrane, which has been proved to be related to cancer metastasis in some studies. Accurate prediction of lymph node metastasis in patients with gastric SRCC is of great significance for effective clinical treatment and ensuring a better prognosis.

### Lymph node involvement and cell morphology of colorectal SRCC

Colorectal SRCC is a rare subtype of colorectal cancer with distinctive molecular characteristics, including a low incidence of KRAS, PIK3CA, and APC mutations^1^. A study shows that diffuse infiltrating colorectal SRCC with little or no extracellular mucin is more invasive and has a worse prognosis than mucin-rich SRCC, which is often accompanied by peritoneal dissemination^15^. This suggests that there is a certain relationship between the morphology of tumor cells in colorectal SRCC and their biological behavior. Compared with gastric SRCC, colorectal SRCC exhibit more aggressive biological behavior and patients were often diagnosed with neoplasms of greater size, which explained the limited number of T1 and T2 cases in our cohort, hence we only analyze the status of lymph node involvement of colorectal SRCC. This part of the analysis cohort consists of 218 HE stained WSIs taken from primary lesions of colorectal SRCC, which are either negative or exhibit metastases in sentinel lymph nodes.

Compared with gastric SRCC, colorectal SRCC has larger SC (*p* < 0.001) and SN (*p* = 0.002), while the NCR was lower (*p* = 0.003). It is worth noting that atypia of colorectal SRCC is more obvious than gastric SRCC which is mainly reflected in SC SD (p < 0.001), SN SD (*p* < 0.001), and EP SD (*p* = 0.031) (Supplementary Table 3). The results are consistent with the objective fact that the prognosis of colorectal SRCC is worse, and further explain its aggressive biological behavior.

**Figure 4:**
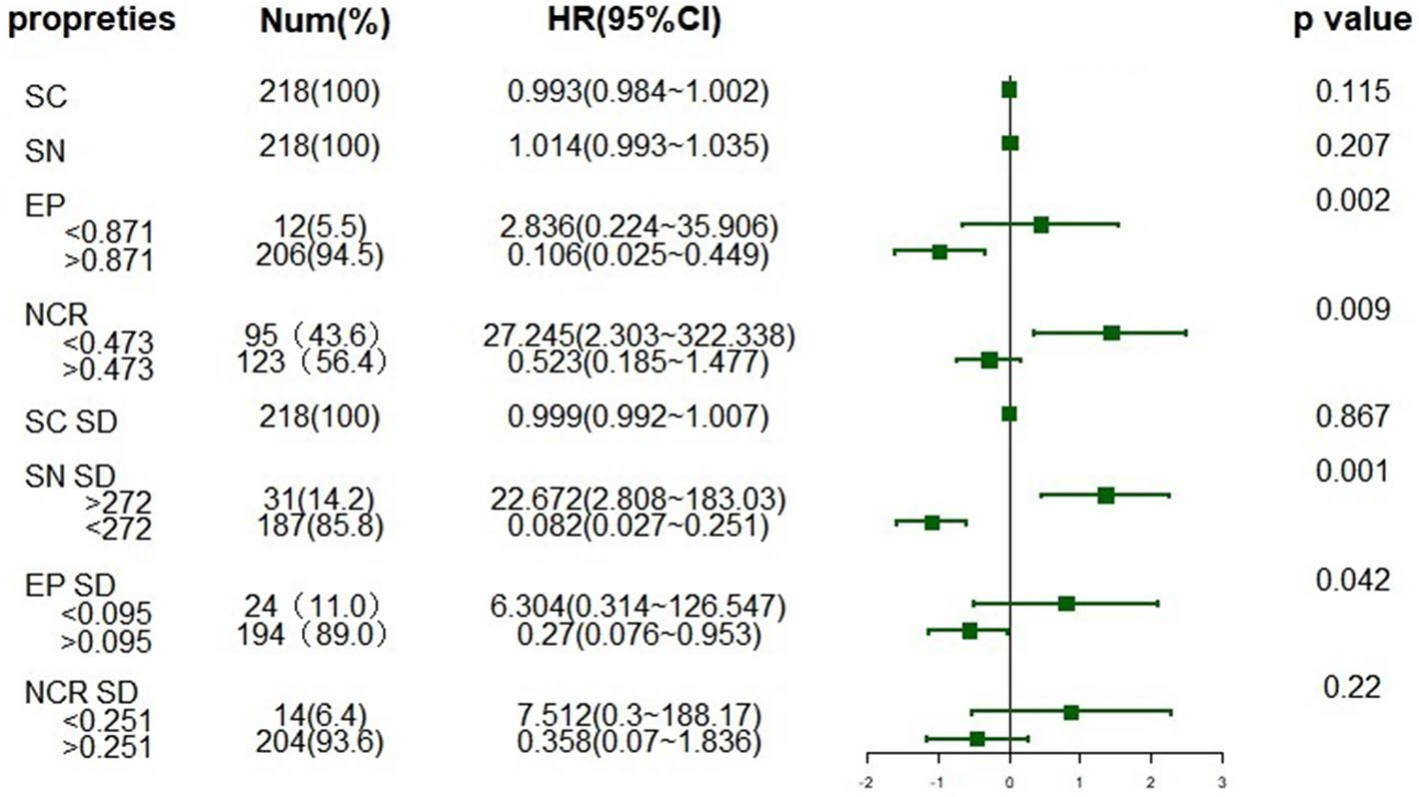
Forest plot of lymph node involvement in colorectal SRCC. The cutoff value of each index is calculated by the Youden's index and its corresponding optimal cutoff point. HR hazard ratio. X axis is scaled by logarithmed HR.

In addition, our results further confirmed some independent significant predictors of lymph node metastasis in colorectal SRCC. We found the SC (*p* = 0.001), SN (*p* = 0.001) and EP (*p* = 0.001) were the most correlated inherent properties with lymph node involvement (Supplementary Table 4). The cases with lymph node involvement have bigger cell and nuclear cross-sectional area than the rests, while the cell ellipticity is relatively smaller. SC SD (*p* = 0.001), SN SD (*p* = 0.001) and are the most related parameters of atypia with lymph node metastasis. Moreover, EP (HR 2.836, 95%CI 0.224–35.906, *p* = 0.002) and SN SD (HR 22.672, 95%CI 2.808–183.03, *p* = 0.001) were identified as independent significant predictors of lymph node metastasis in colorectal SRCC (Fig. 4). Obviously, in cases with lymph node involvement, the atypia of cancer cells is greater in all three categories. When colorectal SRCC lymph nodes are involved, the tumor cells have greater sizes with enlarged, irregular-shaped nuclei. The results show that we can predict the metastatic potential of different cases from the morphology of signet ring cells. Studies have shown that in many types of cancer, compared with non-metastatic cells, the membrane of metastatic cells tends to be softer mechanically, and there are common changes in other physical properties, such as traction, migration behavior, and mechanical stiffness. Tumor cells’ transformation through accumulating mutations, leading to a complex and highly heterogeneous of morphological characteristics and physical properties^16^.

### Different forms of metastatic lesions

It is well known that in addition to the higher rate of lymph node metastasis, SRCC also has several other paths of metastasis. Peritoneal dissemination is a classic metastasis form of gastrointestinal cancer. The "seed and soil" theory has been established as the basic theory^17^. Besides, about 50% of the metastatic tumors of the ovary are Krukenberg tumors, most of them came from SRCC^18^. Gastric SRCC is the most common primary tumor, followed by colorectal cancer. It is generally believed that the tumor cells in the metastatic lesion are one or more subclones in the primary lesion which is competent to overcome the metastatic barriers^16^. To metastasize, a cell must overcome multiple obstacles in the metastatic cascade—invasion and migration through the stromal, vessels, survival from shear forces of blood flow, successful re-attachment to blood vessel walls, and thus settle down into another place^17^. Different metastatic lesions from the same tumor need to face distinct metastatic barriers, its internal molecular changes are diverse, and the prognostic significance is also different—these are directly associated with the cellular physical properties, such as the formation and destruction of the cytoskeleton^19^, the process of epithelial-mesenchymal transition^20^, or the effect of extracellular matrix on cells^21^, which are all directly related to cell morphology.

Therefore, to explore the differences among metastatic lesions, we analyzed the metastatic regional lymph nodes, peritoneal implants, and Krukenburg tumors by DL. The differences in cellular properties and atypia among the three categories are listed in Supplementary Table 5.

In terms of SC and its atypia, peritoneal implants were the largest, Krukenburg tumors were the second, and regional lymph nodes were the smallest (*p* < 0.01). Metastatic regional lymph nodes are so different from peritoneal implants that all four cellular properties show a significant difference. Peritoneal implants have bigger SC (*p* < 0.001) and SN (*p* < 0.001) than regional lymph nodes and smaller EP (*p* = 0.049) and NCR (*p* = 0.012). Krukenburg tumor shows less atypia than regional lymph nodes (*p* = 0.031) and peritoneal implants (*p* = 0.011) in the degree of EP, and less atypia than peritoneal implants in the degree of SN (*p* = 0.016). The atypia of NCR is more obvious than peritoneal implants (*p* = 0.02). It is easy to notice that the atypia of peritoneal implants is the most prominent, which may mean that there are relatively fewer barriers to metastasis to the peritoneal, and more tumor subclones will be able to meet this condition. Moreover, compared to regional lymph nodes, the difference in cell size of Krukenburg tumors is more obvious, but the shape is relatively more regular. This may indicate that more consideration should be given to whether these genes are located in the molecular pathways that play a major role in cell growth when we look for genes that play an important role in metastasis to the ovary. While the genes that play a more important role in lymph node metastasis may regulate the physical properties of cell membrane fluid^22^, affect the cytoskeletal rearrangements within cancer cells^23^, and drive their invasion and migration through the stroma^24^.

The results of this experiment showed that there were significant differences in cell properties and atypia among metastatic regional lymph nodes, peritoneal implants, and Krukenburg tumors, and further confirmed that different forms of metastatic lesions represented different biological events. A study has analyzed the whole gene expression of gastric cancer cells with different metastatic potential^25^. The results suggest that there is a significant difference in gene expression between gastric cancer cells with abdominal metastasis and lymph node metastasis. The same result has also been confirmed by experiments in other common tumors, such as breast cancer and pancreas cancer^26^. These studies are carried out on cell lineages, and the biggest limitation is that they can not reflect the most real biological behavior of tumors objectively in a variety of complex body environments. However, our study makes up for the above regret.

## Discussion

Whether SRCC is an independent tumor entity has been questioned for a long, because of its vague definition and variable prognosis. It is challenging to predict biological behavior and formulate accurate treatment due to the limitations of traditional pathological examination^1–3,14^. However, pathologists could only visually detect signet ring cells under the microscope, and carry out limited qualitative analysis and inaccurate quantitative analysis. Several deep learning models for the diagnose of gastrointestinal tract cancers based on WSIs have been reported. The advance functions including automated histological classification^4,26,27^, immunohisto- chemistry results interpretation^28^, or even microsatellite instability prediction^29^. However, these studies are based on histology and do not perform the identification of individual tumor cells. Moreover, all of these studies acknowledge that there are some deficiencies in their diagnosis of poorly differentiated cancers, including SRCC, because they do not have complex histological structures such as glands or papillaries that can be identified in well-differentiated adenocarcinomas.

For the first time, we used DL to analyze the morphology of gastrointestinal SRCC and its different metastatic lesions (lymph nodes, peritoneal implants, and Kukenburg tumors) and accurately measure the inherent properties of signet ring cells, including the cross-sectional area of cell plasma and nuclear, the cell ellipticity, as well as the nuclear/cytoplasmic ratio. We analyzed these inherent properties and used them to define different dimensions of cell atypia. As is known to all, cell morphology is the macroscopic expression of the final coding proteins by the tumor genome^30^. Compared with the analysis of some specific genes, cell morphology is relatively easier to observe and measure^31^. A tumor with greater heterogeneity, or an "ugly tumor", usually has a worse prognosis, which has been widely recognized by pathologists. The measurement of intra-tumor heterogeneity can be used as a biomarker to predict treatment and improve outcome^32^, while the same picture can also be captured by DL to achieve a better prediction effect^33,34^. Compared with previous studies on SRCC, our results quantify the specific manifestations of atypia, or how ugly the tumor is, in more detail, and clarify that cell morphology reflects the outcome of the tumor objectively. Our results provide new insight into the biological processes involved in disease etiology and can be used as biomarkers for the diagnosis or prognosis of SRCC.

Our research has the potential to be further developed. The research was conducted by Sundar et al.^35^ on primary gastric cancer intratumoral heterogeneity has revealed the significant differences in gene expression in tumor superficial areas, deep areas, and regional lymph node from the gene level, which may affect the treatment. Therefore, we're indicated to concentrate our future work on evaluating the multi-dimensional information of different subregions in the primary lesion and develop appropriate segmentation algorithms to confirm the impact of temporal and spatial heterogeneity of the tumor at the cell level. In addition, cell morphology reflects the complex genomic and gene expression changes of cancer cells, and the measurement of morphological heterogeneity combined with functional spectrum can be a powerful, high-throughput, economical and effective means to diagnose and guide treatment. Therefore, we can further explore and clarify the relationship between the morphology and molecular changes of SRCC tumor cells. In addition, we will try to establish a prognosis cohort of different patients from different regions to confirm whether our algorithm can predict prognosis or guide treatment as good as or better than the existing tumor staging system.

As more and more data being available and algorithms become perfect, we hope that our results can be combined with biopsy, pathological examination, gene sequencing, and other means of testing methods, which will contribute to more effective stratified treatment, survival prediction, and patient management, and improve the treatment decisions and outcomes of SRCC patients.

## Methods

### Datasets information

Each case of our data set was prepared in the Department of Pathology, Ruijin Hospital, Shanghai Jiaotong University School of Medicine in 2014–2020. The WSIs were produced at × 40 magnification (0.238 μm/pixel) by the National Medical Products Administration-cleared KFBio KF-PRO-005 digital scanner. All of the sections were evaluated by two pathologists and reviewed by a senior pathologist through a standardized procedure. We adopt the 4th edition of the WHO Classification of Tumors of the Digestive System as the reference standard. The patients who received neoadjuvant chemotherapy were excluded to eliminate the effect of treatment on cell morphology.

For gastric and colorectal SRCC, we selected 1–4 HE sections of each case, which was determined by the number of tumor cells at the time of initial evaluation. In order to ensure the stability of the test, sections containing at least 500 signet ring cells will be selected. We also selected all the Krukenburg tumors and peritoneal implants of SRCC in our case bank over the past six years. Cases with poor image quality were excluded. Additional clinical information was listed (Supplementary Table 1).

### Statistical information

Statistical analysis was conducted by IBM SPSS (Release 25.0) and R ×64 4.0.3, *P*-value < 0.05 was considered statistically significant. Figures were then subsequently edited by Adobe Photoshop CS6 and GraphPad Prism 8 to provide better clarity. Continuous value variables such as the relationship between the parameters and lymph node involvement were analyzed by independent sample T-test and Mann–Whitney U test, nonparametric test and pairwise comparison of multiple independent samples were analyzed by Kruskal–Wallis test and Tukey HSD test. Binary logistic regression was used to compute the hazard ratio (HR) with 95% CI of patients with lymph node involvement in colorectal SRCC. Youden index was used in calculating its corresponding optimal cutoff point.

### Signet ring cell and nuclei segmentation

The deep learning methodology was conducted by Pytorch (Release 1.1.0) to perform Signet Ring Cell and Nuclei segmentation. The architecture of the deep learning model is called Deep Layer Aggregation^9^, a variant to UNet, which is an elaborately designed connection manner of convolution modules to assemble image features from various scales. A coarse UNet was established to find an approximate region that contains Signet Ring Cell at X10 magnification, to swiftly discard most of the benign parts in whole slide images. We developed another fine UNet to carry out four class segmentation for the constant size of an image input in 512 pixels height and width, at X40 magnification, who classifies each pixel to the background, cell, nuclei, or instance boundary. To extend the model robustness on the various source of whole slide images, while it is unattainable to make pixel-level annotations to numerous images, a semi-supervised learning methodology was conducted as described in Li et al.^11^, for models running on X40 magnification.

For each independent Signet Ring Cell, the nucleus which has the largest intersection area is assigned to this cell. Then those morphological features of each cell could be easily obtained based on precise cell and nuclei mask, with the help of Opencv (2.4.9) and Scikit-Image (0.17.2). Based on thousands of cells will we have statistical results for each whole slide image for analysis. In details, based on the exact mask of cells and nuclei.

Cell Area is determined as the total number of pixels of Signet Ring Cell instance mask, Nucleus Area is the number of pixels of Nucleus instance mask. The aspect ratio of the minimum area rotated bounding box of each SRCC instance mask is called Cell Aspect Ratio. Nuclear Cytoplasmic Ratio is defined as the Nucleus Area divided by Cell Area.

Signet ring cell detection algorithms were developed by Pytorch1.1.0 and Python3.6.1. Thumbnail image and patches at 0.25 μm are extracted from whole slide image by Openslide3.4.1. After detecting cells at 0.25 μm, we downscale cell coordinates into a mask whose size is same as thumbnail image to generate gaussian map, pixel values near by cell coordinates are higher. With such gaussian map we could generate heatmap, then merge together with the thumbnail image to be those in Fig. 2.

### Inference status

Previously as described in our former work^11^, during three folds cross-validation, there exists obvious scores degrading from easy (Ins Recall 0.705, Nor FPs 1.45, Ins FROC 0.692) separation to hard (Ins Recall 0.658, Nor FPs 0.943, Ins FROC 0.657) separation for not enough training data. In this paper we add extra 1000 unlabeled patches containing crowded signet ring cell and 2000 benign patches into the cooperative training procedure, leading to similar scores between easy (Ins Recall 0.713, Nor FPs 0.912, Ins FROC 0.702) and hard (Ins Recall 0.709, Nor FPs 0.904, Ins FROC 0.695) mode. With similar scores between the two data separation modes, we believe the model generalization could be enough for new whole slide images. By introducing a coarse signet ring cell detection model on X10 magnification to discard most of the benign regions, averagely for each whole slide image inference time is reduced from 20 to 3 min, compared to predicting all the areas on × 40 magnification.

#FROC: by adjusting the confidence threshold, when the number of normal region's false positives is 1, 2, 4, 8, 16, 32, the FROC is the average of relevant recall at those confidence thresholds. Ins Recall: instance-level recall, Nor FPs: normal region false positives.

### Ethical statements

This study did not involve human trials or participants, so it did not require approval from ethical committee.

## Supplementary Information

Supplementary Information.

## References

[1] Korphaisarn K (2019). Signet ring cell colorectal cancer: genomic insights into a rare subpopulation of colorectal adenocarcinoma. Br J Cancer.

[2] Pokala SK (2018). Incidence, survival, and predictors of lymph node involvement in early-stage gastric signet ring cell carcinoma in the US. J. Gastrointest. Surg..

[3] Pernot S (2015). Signet-ring cell carcinoma of the stomach: Impact on prognosis and specific therapeutic challenge. World J. Gastroenterol..

[4] Song Z (2020). Clinically applicable histopathological diagnosis system for gastric cancer detection using deep learning. Nat. Commun..

[5] Campanella G (2019). Clinical-grade computational pathology using weakly supervised deep learning on whole slide images. Nat. Med..

[6] Levine AB (2019). Rise of the machines: advances in deep learning for cancer diagnosis. Trends in Cancer.

[7] Kermany DS (2018). Identifying medical diagnoses and treatable diseases by image-based deep learning. Cell.

[8] Falk T (2019). U-Net: deep learning for cell counting, detection, and morphometry. Nat. Methods.

[9] Yu, F. *et al*. Deep layer aggregation. in 2018 IEEE/CVF Conference on Computer Vision and Pattern Recognition. (2018).

[10] Wang Y (2019). Deep-learning-based polar-body detection for automatic cell manipulation. Micromachines (Basel).

[11] Li J (2019). Signet ring cell detection with a semi-supervised learning framework. International conference on information processing in medical imaging.

[12] Hasuike N (2018). A non-randomized confirmatory trial of an expanded indication for endoscopic submucosal dissection for intestinal-type gastric cancer (cT1a): the Japan Clinical Oncology Group study (JCOG0607). Gastric Cancer.

[13] Phillip JM (2021). A robust unsupervised machine-learning method to quantify the morphological heterogeneity of cells and nuclei. Nat. Protoc..

[14] Miyashiro I (2014). High false-negative proportion of intraoperative histological examination as a serious problem for clinical application of sentinel node biopsy for early gastric cancer: final results of the Japan Clinical Oncology Group multicenter trial JCOG0302. Gastric Cancer.

[15] Hartman DJ (2013). Signet ring cell colorectal carcinoma: a distinct subset of mucin-poor microsatellite-stable signet ring cell carcinoma associated with dismal prognosis. Am. J. Surg. Pathol..

[16] Wu P (2016). Evolution of cellular morpho-phenotypes in cancer metastasis. Sci. Rep..

[17] Sun F, Feng M, Guan W (2017). Mechanisms of peritoneal dissemination in gastric cancer. Oncol. Lett..

[18] Zulfiqar M (2020). Krukenberg tumors: update on imaging and clinical features. AJR Am. J. Roentgenol..

[19] Fife CM, McCarroll JA, Kavallaris M (2014). Movers and shakers: cell cytoskeleton in cancer metastasis. Br. J. Pharmacol..

[20] Aiello NM, Kang Y (2019). Context-dependent EMT programs in cancer metastasis. J. Exp. Med..

[21] Yuzhalin AE (2018). Dynamic matrisome: ECM remodeling factors licensing cancer progression and metastasis. Biochim. Biophys. Acta (BBA) Rev. Cancer.

[22] Nicolson GL (2015). Cell membrane fluid-mosaic structure and cancer metastasis. Cancer Res..

[23] Hall A (2009). The cytoskeleton and cancer. Cancer Metastasis Rev..

[24] Massagué J, Obenauf AC (2016). Metastatic colonization by circulating tumour cells. Nature.

[25] Hippo Y (2001). Differential gene expression profiles of scirrhous gastric cancer cells with high metastatic potential to peritoneum or lymph nodes. Cancer Res..

[26] Iizuka O (2020). Deep learning models for histopathological classification of gastric and colonic epithelial tumours. Sci. Rep..

[27] Yoshida H (2018). Automated histological classification of whole-slide images of gastric biopsy specimens. Gastric Cancer.

[28] Sharma H (2017). Deep convolutional neural networks for automatic classification of gastric carcinoma using whole slide images in digital histopathology. Comput. Med. Imaging Graph.

[29] Kather JN (2019). Deep learning can predict microsatellite instability directly from histology in gastrointestinal cancer. Nat. Med..

[30] Wu PH (2015). Evolution of cellular morpho-phenotypes in cancer metastasis. Sci. Rep..

[31] Wu P (2020). Single-cell morphology encodes metastatic potential. Sci. Adv..

[32] Almendro V, Marusyk A, Polyak K (2013). Cellular heterogeneity and molecular evolution in cancer. Ann. Rev. Pathol..

[33] Kulkarni PM (2020). Deep learning based on standard h&e images of primary melanoma tumors identifies patients at risk for visceral recurrence and death. Clin. Cancer Res..

[34] Courtiol P (2019). Deep learning-based classification of mesothelioma improves prediction of patient outcome. Nat. Med..

[35] Sundar R (2020). Spatial profiling of gastric cancer patient-matched primary and locoregional metastases reveals principles of tumour dissemination. Gut.

