# Supplementary material for: Quantifying the cell morphology and predicting biological behavior of signet ring cell carcinoma using deep learning
Source: Sci Rep. 2022 Jan 7;12:183. doi: 10.1038/s41598-021-03984-4 (PMC8741938; doi:10.1038/s41598-021-03984-4)
Supplement: Supplementary file 1 — Supplementary Information. [file 41598_2021_3984_MOESM1_ESM.docx]

| **Supplementary table 1 Descriptive statistics of the 607 WSIs** | | | | | | | | | | | | | | | |
| --- | --- | --- | --- | --- | --- | --- | --- | --- | --- | --- | --- | --- | --- | --- | --- |
| Lesion | | | Number of WSIs (%) | | | Cell num. | SC | SN | Ep | NCR | SC SD | SN SD | Ep SD | NCR SD | |
| **Stomach** | | | 259(42.7) | | | 10,929,889 | 955.8 | 388.4 | 0.8886 | 0.4683 | 723.0 | 316.4 | 0.1015 | 0.3008 | |
|  | primary lesion | | | 87(33.6) | | 3,302,359 | 818.3 | 404.3 | 0.8858 | 0.5519 | 570.4 | 311.6 | 0.1003 | 0.2857 | |
|  | T stage | | |  | |  |  |  |  |  |  |  |  |  | |
|  | | T1 | | | 13(14.9) | 50,873 | 918.0 | 286.3 | 0.8863 | 0.3855 | 695.1 | 246.9 | 0.1013 | 0.2955 | |
|  | | T2 | | | 17(19.5) | 192,774 | 741.4 | 344.5 | 0.8884 | 0.5147 | 523.5 | 281.1 | 0.0991 | 0.3101 | |
|  | | T3 | | | 29(33.3) | 1,357,711 | 795.2 | 385.1 | 0.8862 | 0.5368 | 538.3 | 281.0 | 0.0993 | 0.2938 | |
|  | | T4 | | | 28(32.2) | 1,701,001 | 842.6 | 515.2 | 0.8837 | 0.6672 | 574.2 | 392.0 | 0.1016 | 0.2580 | |
|  | N stage | | | |  |  |  |  |  |  |  |  |  |  | |
|  | | negative | | | 38(43.7) | 1,392,866 | 795.8 | 406.6 | 0.8871 | 0.5587 | 541.6 | 311.6 | 0.0991 | 0.2849 | |
|  | | positive | | | 49(56.3) | 1,909,493 | 835.8 | 402.5 | 0.8848 | 0.5466 | 592.7 | 311.7 | 0.1013 | 0.2863 | |
|  | lymph nodes | | | 74(28.6) | | 1,239,012 | 798.8 | 359.2 | 0.8911 | 0.4699 | 607.1 | 285.6 | 0.1042 | 0.3021 | |
|  | peritoneal implants | | | 33(12.7) | | 281,126 | 1283.1 | 403.6 | 0.8901 | 0.3680 | 1084.7 | 351.2 | 0.1048 | 0.3100 | |
|  | Krukenburg tumor | | | 65(25.1) | | 6,107,392 | 1152.5 | 392.6 | 0.8887 | 0.4055 | 875.5 | 340.2 | 0.0983 | | 0.3148 |
| **Colorectum** | | | 348(56.8) | | | 18,512,930 | 1118.6 | 445.5 | 0.8860 | 0.4619 | 874.9 | 435.6 | 0.1023 | 0.3000 | |
|  | primary lesion | | | 218(62.6) | | 14,102,513 | 1125.2 | 465.9 | 0.8837 | 0.4848 | 877.1 | 448.4 | 0.1030 | 0.2981 | |
|  | T stage | | |  | |  |  |  |  |  |  |  |  |  | |
|  | | T1 | | | 4(6.5) | 77,383 | 1173.6 | 467.5 | 0.8728 | 0.4681 | 929.2 | 427.3 | 0.1090 | 0.2929 | |
|  | | T3 | | | 69(31.7) | 3,231,069 | 1179.8 | 491.7 | 0.8828 | 0.4864 | 946.2 | 519.0 | 0.1038 | 0.3133 | |
|  | | T4 | | | 145(66.5) | 10,794,061 | 1097.9 | 453.5 | 0.8844 | 0.4845 | 843.3 | 415.5 | 0.1024 | 0.2910 | |
|  | N stage | | | |  |  |  |  |  |  |  |  |  |  | |
|  | | negative | | | 31(14.2) | 2,818,987 | 967.6 | 391.2 | 0.8888 | 0.4973 | 714.1 | 335.4 | 0.1010 | 0.2931 | |
|  | | positive | | | 187(85.8) | 11,283,516 | 1151.3 | 478.2 | 0.8828 | 0.482 | 904.2 | 467.2 | 0.1033 | 0.2989 | |
|  | lymph nodes | | | 105(30.2) | | 4,056,993 | 1024.8 | 385.4 | 0.8912 | 0.4283 | 810.0 | 382.3 | 0.1009 | 0.3085 | |
|  | peritoneal implants | | | 25(7.2) | | 353,424 | 1455.8 | 520.1 | 0.8844 | 0.4029 | 1128.4 | 547.9 | 0.1028 | 0.2811 | |
| **total** | | | | 607(100) | | 29,442,819 | 1049.2 | 421.1 | 0.8871 | 0.4646 | 810.1 | 384.8 | 0.1020 | 0.3003 | |

SC Cell area (pixel); SN Nucleus area (pixel); Ep Ellipticity; NCR Nuclear-plasma ratio; SD Standard deviation

| **Supplementary table 2** Analysis of lymph node involvement in gastric SRCC | | | |
| --- | --- | --- | --- |
|  | positive | negative | P value |
| SC | 835.77(626.24~1045.31) | 795.76(607.40~984.11) | 0.359 |
| SN | 402.46(265.80~539.13) | 406.65(294.12~519.19) | 0.879 |
| EP | 0.8848(0.8775~0.8921) | 0.8871(0.8784~0.8958) | 0.179 |
| NCR | 0.5466(0.3795~0.7137) | 0.5587(0.4120~0.7054) | 0.726 |
| SC SD | 592.75(387.86~797.63) | 541.58(338.85~744.31) | 0.249 |
| SN SD | 311.71(191.65~431.78) | 311.55(210.98~412.12) | 0.995 |
| EP SD | 0.1013(0.0967~0.1058) | 0.0991(0.0935~0.1047) | **0.045** |
| NCR SD | 0.2863(0.2423~0.3303) | 0.2849(0.2567~0.3131) | 0.871 |

| **Supplementary table 3** Inherent property and atypia of solid mass | | | | | | | | | |
| --- | --- | --- | --- | --- | --- | --- | --- | --- | --- |
|  | **Stomach-Colorectum** | | | **Stomach-Krukenburg tumor** | | | **Colorectum -Krukenburg tumor** | | |
|  | Stomach | Colorectum | p value | Stomach | Krukenburg | p value | Colorectum | Krukenburg | p value |
| SC | 818.3 | 1125.2 | **<0.001** | 818.3 | 1152.5 | **0.001** | 1125.2 | 1152.5 | 0.79 |
| SN | 404.3 | 465.9 | **0.002** | 404.3 | 392.6 | 0.845 | 465.9 | 392.6 | **<0.001** |
| Ep | 0.8858 | 0.8837 | 0.257 | 0.8858 | 0.8887 | 0.064 | 0.8837 | 0.8887 | **<0.001** |
| NCR | 0.5519 | 0.4848 | **0.003** | 0.5519 | 0.4055 | **0.001** | 0.4848 | 0.4055 | **<0.001** |
| SC SD | 570.4 | 877.1 | **<0.001** | 570.4 | 875.5 | **0.001** | 877.1 | 875.5 | 1.0 |
| SN SD | 311.6 | 448.4 | **<0.001** | 311.6 | 340.2 | 0.806 | 448.4 | 340.2 | **<0.001** |
| Ep SD | 0.1003 | 0.1030 | **0.031** | 0.1003 | 0.0983 | 0.047 | 0.1030 | 0.0983 | **<0.001** |
| NCR SD | 0.2857 | 0.2981 | 0.058 | 0.2857 | 0.3148 | **0.001** | 0.2981 | 0.3148 | **0.002** |

| **Supplementary table 4**  Analysis of lymph node involvement in colorectal SRCC | | | |
| --- | --- | --- | --- |
|  | Positive | Negative | P value |
| SC | 1151.30(884.01~1418.59) | 967.59(659.40~1275.78) | **<0.001** |
| SN | 478.23(340.71~615.75) | 391.21(292.75~489.67) | **<0.001** |
| EP | 0.8828(0.8752~0.8905) | 0.8888(0.8746~0.9030) | **<0.001** |
| NCR | 0.4827(0.36127~0.60413) | 0.4973(0.3650~0.6297) | 0.543 |
| SC.SD | 904.18(633.93~1174.43) | 714.08(405.18~1022.97) | **0.001** |
| SN.SD | 467.18(276.6063~657.7527) | 335.38(196.50~474.27) | **0.001** |
| EP.SD | 0.1033(0.0983~0.1083) | 0.1010(0.0864~0.1156) | 0.098 |
| NCR.SD | 0.2989(0.2692~0.3286) | 0.2931(0.2579~0.3283) | 0.33 |

| **Supplementary table 5 Univariate analysis of metastatic tumor** | | | | | | | | | | |
| --- | --- | --- | --- | --- | --- | --- | --- | --- | --- | --- |
|  |  | Krukenburg tumor - Peritoneal implants | | | LN - Peritoneal implants | | | Krukenburg tumor - LN | | |
|  |  | Krukenburg tumor | Peritoneal implants | p value | LN | Peritoneal implants | p value | Krukenburg tumor | LN | p value |
| Inherent property | SC | 1152.5 | 1357.6 | **0.0016** | 930.9 | 1357.6 | **<0.001** | 1152.5 | 930.9 | **<0.001** |
|  | SN | 392.6 | 453.8 | **0.049** | 374.5 | 453.8 | **<0.001** | 392.6 | 374.5 | 0.66 |
|  | Ep | 0.8887 | 0.8876 | 0.83 | 0.8913 | 0.8876 | **0.049** | 0.8887 | 0.8913 | 0.19 |
|  | NCR | 0.4055 | 0.3830 | 0.67 | 0.4461 | 0.3830 | **0.012** | 0.4055 | 0.4461 | 0.13 |
| Atypia | SC SD | 875.5 | 1103.5 | **<0.001** | 724.7 | 1103.5 | **<0.001** | 875.5 | 724.7 | **0.008** |
|  | SN SD | 340.2 | 436.0 | **0.016** | 343.0 | 436.0 | **0.004** | 340.2 | 343.0 | 0.99 |
|  | Ep SD | 0.0983 | 0.1039 | **0.011** | 0.1023 | 0.1039 | 0.56 | 0.0983 | 0.1023 | **0.031** |
|  | NCR SD | 0.3148 | 0.2975 | **0.02** | 0.3054 | 0.2975 | 0.31 | 0.3148 | 0.3054 | 0.16 |
